# Epithelial-mesenchymal transition markers screened in a cell-based model and validated in lung adenocarcinoma

**DOI:** 10.1186/s12885-019-5885-9

**Published:** 2019-07-11

**Authors:** Jing Song, Wenqing Wang, Yingyan Wang, Yongxin Qin, Yingzi Wang, Jian Zhou, Xuelian Wang, Yi Zhang, Qi Wang

**Affiliations:** 1 Department of Respiratory Medicine, The Second Hospital, Dalian Medical University, No. 467 Zhongshan Road, Dalian, 116000 Liaoning China; 2 Center for Genome Analysis, ABLife Inc., Optics Valley International Biomedical Park, Building 9-4, East Lake High-Tech Development Zone, 388 Gaoxin 2nd Road, Wuhan, 430075 Hubei China; 3 Laboratory for Genome Regulation and Human Health, ABLife Inc., Optics Valley International Biomedical Park, Building 9-4, East Lake High-Tech Development Zone, 388 Gaoxin 2nd Road, Wuhan, 430075 Hubei China; 4 Laboratory Center for Diagnostics, Dalian Medical University, No. 9 West Section Lvshun South Road, Dalian, 116044 Liaoning China; 5 Department of Critical Care Medicine, The First Hospital, Dalian Medical University, No. 222 Zhongshan Road, Dalian, 116000 Liaoning China

**Keywords:** EMT, Lung adenocarcinoma, RNA-seq, miRNA-seq, WGCNA

## Abstract

**Background:**

Re-capture of the differences between tumor and normal tissues observed at the patient level in cell cultures and animal models is critical for applications of these cancer-related differences. The epithelial-mesenchymal transition (EMT) process is essential for tumor migratory and invasive capabilities. Although plenty of EMT markers are revealed, molecular features during the early stages of EMT are poorly understood.

**Methods:**

A cell-based model to induce lung cell (A549) EMT using conditioned medium of in vitro cancer activated fibroblast (WI38) was established. High-throughput sequencing methods, including RNA-seq and miRNA-seq, and advanced bioinformatics methods were used to explore the transcriptome profile transitions accompanying the progression of EMT. We validated our findings with experimental techniques including transwell and immunofluorescence assay, as well as the TCGA data.

**Results:**

We have constructed an in vitro cell model to mimic the EMT in patients. We discovered that several new transcription factors were among the early genes (3 h) to respond to cancer micro-environmental cues which could play critical roles in triggering further EMT signals. The early EMT markers also included genes encoding membrane transporters and blood coagulation function. Three of the nine-examined early EMT hallmark genes, GALNT6, SPARC and HES7, were up-regulated specifically in the early stages of lung adenocarcinoma (LUAD) and confirmed by TCGA patient transcriptome data. In addition, we showed that miR-3613, a regulator of EGFR pathway genes, was constantly repressed during EMT progress and indicative of an epithelial miRNA marker.

**Conclusions:**

The CAF-stimulated EMT cell model may recapture some of the molecular changes during EMT progression in clinical patients. The identified early EMT hallmark genes GALNT6, SPARC and HES7and miR-3613 provide new markers and therapeutic targets for LUAD for the further clinical diagnosis and drug screening.

**Electronic supplementary material:**

The online version of this article (10.1186/s12885-019-5885-9) contains supplementary material, which is available to authorized users.

## Background

Tumor growth is not only determined by cancer cells proliferation, but also relies on tumor environment, which recently was considered as a target for new anti-metastatic therapies [1]. A subpopulation of cancer adjacent fibroblast can be activated by a diverse set of growth factors secreted from cancer cells [2–4]. The activated fibroblasts, termed as cancer-associated fibroblasts (CAFs), are the most abundant stromal cells in tumor microenvironment that could secret a wide spectrum of chemokines and cytokines into the invasive margins of desmoplastic cancers to promote tumor growth and progression [5–11]. Epithelial-mesenchymal transition (EMT) is a reversible biological process indispensable for development [12]. EMT is reactivated during cancer progression [12–16], which includes initiation, primary tumor growth, invasion, dissemination and metastasis to colonization, as well as acquisition of therapeutic resistance [17–19]. CAFs have been reported to stimulate cancer EMT by activating cellular signaling pathways that increase the invasive features of cancer cells [20–22].

Cell-based models are widely used for EMT studies [23, 24]. Cytokines such as transforming growth factor (TGF)-β are frequently applied for inducing EMT of various epithelial cell types [25, 26]. Besides, conditioned medium of cultured CAFs from cancer tissues of patients have been collected to induce EMT states of epithelial cells [27–29]. In recent years, the rapid accumulation of genome-wide data enabling direct comparisons between the disease and control samples, such as TCGA database, has created an unprecedented opportunity for identification of potential biomarkers and therapeutic targets for cancers [30, 31]. A combination of TCGA- and cell-based screening should expedite the translational medicine process.

To explore this possibility, we co-cultured A549 and WI-38 cells, and then the medium of WI-38 fibroblasts was collected for A549 EMT induction, mimicking the condition of CAF-induced EMT as previously reported [32]. We found that the prototypical EMT markers, the induction of vimentin and repression of E-cadherin were both present in the induced A549 cells. We then gained a comprehensive view of the transcriptomic changes of lung cancer cells during EMT by applying RNA-seq and microRNA-seq (miRNA-seq). The two co-expression modules of genes were specifically upregulated and one miRNA was constantly downregulated at early EMT stages, providing potential biomarkers and therapeutic targets. By analyzing LUAD dataset from TCGA, we found three EMT markers (GALNT6, SPARC and HES7) among the nine in vitro identified genes with known function are also up-regulated in specifically in early stage lung adenocarcinoma patients. These results support the biological relevance of our cell-based screening model for future study of early EMT mechanism and biomarkers, and possibly for drug screening.

## Methods

### Cell culture

Human LUAG A549 cells (CRM-CCL-185) and human lung fibroblast WI38 cells (CCL-75) were obtained from American Type Culture Collection (Manassas, VA, USA) in 2013. These cell lines have been authenticated by short-tandem repeat analyses. They are free of mycoplasma contamination. A549 cells were cultured in RPMI-1640 medium (Gibco, Long Islands, NY), while WI38 cells were cultured in IMDM (Gibco) at 37 °C in a humidified atmosphere of 5% CO_2_. These cell culture media were also supplemented with 10% fetal bovine serum (Hyclone, Logan, UT, USA),penicillin (100 U/mL), and streptomycin (100 μg/mL).

### Tumor cell Transwell invasion assay

Appropriate matrigel (Corning) was used to pre-coated the filters with 8-μm pore size between the upper and bottom chambers of the Transwell apparatus (Corning). After the matrigel solidified at 37 °C overnight, A549 cells were seeded into the upper chambers and then control medium and CAF conditional medium were added into the bottom Transwell chamber and cells were incubated at 37 °C for different time points. Cells on the upper chambers were fixed with 100% methanol for 20 min, stained in DAPI (Sigma) for 10 min and washed with PBS. Cells remaining on the surface of the filter were swabbed with a cotton swab. The number of cells invaded into the lower surface of the polycarbonate filter was counted at 100× magnification under a light microscope.

### Immunofluorescence assay

For immunofluorescence staining, cells were grown on a Glass Bottom Cell Culture Dish (Nest, Wuxi, China) until 50–60% confluence, fixed with 4% paraformaldehyde and permeabilized with 0.3% Triton X-100. After washing three times with cold PBS, cells were incubated with anti-E-cadherin (Invitrogen, Carlsbad, USA) and anti-Vimentin antibodies (Abcam, Cambridge, UK) at 4 °C for one hour, followed by Alexa Fluor 488-labeled and 594-labeled secondary antibody (Proteintech, Wuhan, China) for one hour, and counterstained with DAPI (Sigma, St Louis, USA). Images were subsequently captured using a confocal microscope (Leica TCS SP5, Mannheim, Germany).

### MiR-3613 mimic experiment

Human LUAG A549 cells were obtained from American Type Culture Collection (Manassas, VA, USA) and cultured in RPMI-1640 medium (Gibco, Long Islands, NY). MiR-3613-3p mimic and corresponding negative control (random sequences) were purchased from GenePharma (Suzhou, China). Cells were transfected with the miR-3613 mimic, negative control (NC) using Lipofectamine 2000 transfection reagent (Invitrogen, Carlsbad, CA, USA). Opti-MEM I Reduced Serum Medium (Gibco, Grand Island, NY, USA) was used to dilute Lipofectamine 2000 and nucleic acids. The detailed sequence information is presented in Additional file 6: Table S1.

### Transcriptome sequencing

Transcriptome sequencing of 12 RNA samples from A549 cells collected at different time points was carried out. Libraries were prepared using RNA-seq Library Preparation Kit for Whole Transcriptome Discovery (Gnomegen), and Balancer NGS Library Preparation Kit for small/microRNA (GnomeGen) following manufacture’s instruction. The libraries were applied to illumina NextSeq 500 system for 151 nt pair-end sequencing by ABlife Inc. (Wuhan, China).

### Data processing

Clean reads were aligned to the human-hg19 genome using TopHat2 [33]. Reads with only one genomic location were preserved for RPKM (reads per kilobase of exon model per million mapped reads) calculation [34]. Differentially expressed genes (DEGs) were analyzed by edgeR [35]. For each gene, the *p*-value was computed and the significance threshold to control FDR at a given value was calculated.

### Weighted gene correlation network analysis (WGCNA)

To get the expression module and distinguish genes from a union set by expression feature, we use the weighted gene correlation network analysis (WGCNA) [36]. RPKM files of DEGs by any pair were used as the input. The output is the gene modules according to their expression pattern. For each gene module, eigengene was chosen to represent the expression pattern.

### Functional enrichment analysis

Gene Ontology (GO) and KEGG enrichment analysis was performed with KOBAS 2.0 [37]. Hypergeometric test was performed with robust FDR correction to obtain an adjusted *P*-value between certain tested gene groups and genes annotated in the reference genome.

### RT-qPCR

Total RNA was prepared from A549 cells with TRIzol Reagent (Life Technology) according to the manufacturer’s instructions. DNA was eliminated by DNase I treatment and RNA were purified by sequential phenol-chloroform extraction and isopropanol precipitation, and dissolved in sterile RNase-free water. Complementary DNA was synthesized from 4 μg total RNA with random hexamers and quantitative real-time PCR analysis was performed with SYBR green real-time PCR mix (Toyobo) in a real-time detection system (Bio-Rad). GAPDH and U6 genes were used as the internal control genes for mRNA and miRNA, respectively. The primers used in this study are listed in Additional file 6: Table S1.

### MiRNA targets prediction

For each miRNA, we predict the target mRNAs using two software, TargetScan (version 7.1) [38] and miRanda (version 3.3) [39] with default parameters. The results from the two methods were combined to generate a complete list of miRNA target genes.

### TCGA data analysis

Transcriptome profiling and clinical data of LUAD patients were downloaded from The Cancer Genome Atlas database (https://www.cancer.gov/about-nci/organization/ccg/research/structural-genomics/tcga). Expression differences between normal and cancer tissues were analyzed using edger [35]. For each gene, the *p*-value was computed and the significance threshold to control FDR at a given value was calculated.

## Results

### Activated WI-38 CM induced A549 cell epithelial-mesenchymal transition

**Fig. 1:**
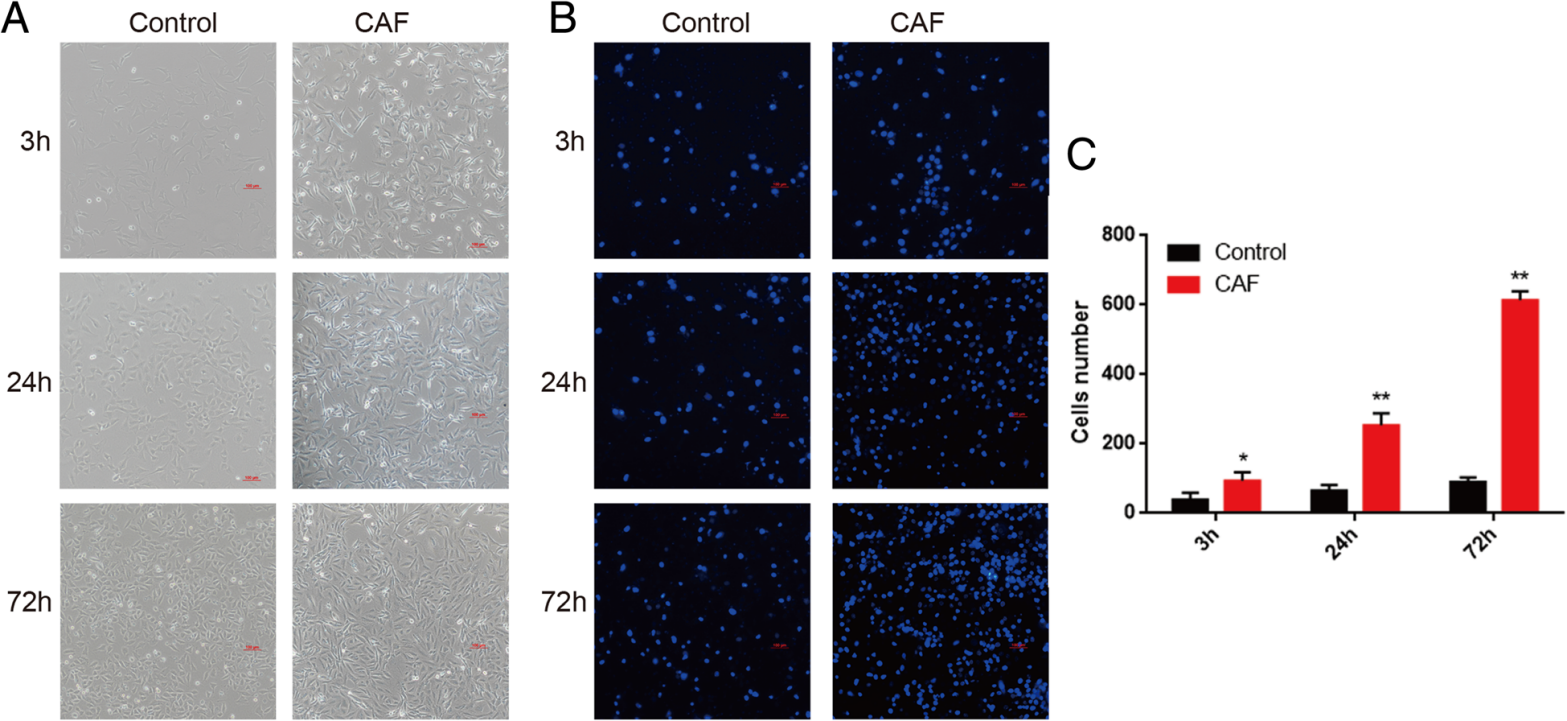
CAF-CM induces mesenchymal phenotypes in A549 cell**. a** Bright-field images of CAF conditioned medium or control treated A549 cells at 3, 24and 72 h. CAF-CM treated A549 cells showed the loss of cell-cell contact and scattering phenotypes at 24 and 72 h, while control cells maintained the epithelial cobblestone shape. **b** DAPI staining images of CAF conditioned and control medium treated A549 cells at 3, 24 and 72 h subject to Transwell matrigel. CAF-CM treated cells showed enhanced migratory and invasive properties than control at 3, 24 and 72 h. **c** Quantitative analysis of Transwell assay showed enhanced migratory and invasive properties after CAF-CM treatment. * *p* < 0.05; ** *p* < 0.01; *** *p* < 0.001; t-test

To closely monitor the progress of EMT, we aimed to develop an in vitro system to induce EMT using CAF conditioned medium (CAF-CM). The conditioned medium of CAF cell culture from cancer patients has been used [28], and we asked whether CAF can be obtained from in vitro as well. We used the CM from human lung adenocarcinoma A549 cell culture to activate human fetal lung fibroblast WI-38 cells for 24 h, and then collected the medium to induce A549 EMT. To investigate whether the activated WI-38 conditioned medium mimics tumor microenvironment, we collected A549 cells incubated with the conditioned and control medium at various time points to check EMT related phenotypes. After 3 h of treatment, CAF-CM treated A549 cells showed morphological change and enhanced invasive ability compared with control cells (Fig. 1a and b). Distinct mesenchymal phenotypes were consistently observed after 24 and 72 h treatment, when A549 cells acquired an elongated and scattered cell shape (Fig. 1a), and also displayed higher invasion through Transwell matrigel (Fig. 1b and c). These results suggest that the activated WI-38 conditioned medium is effective to induce EMT in A549 cells.

**Fig. 2:**
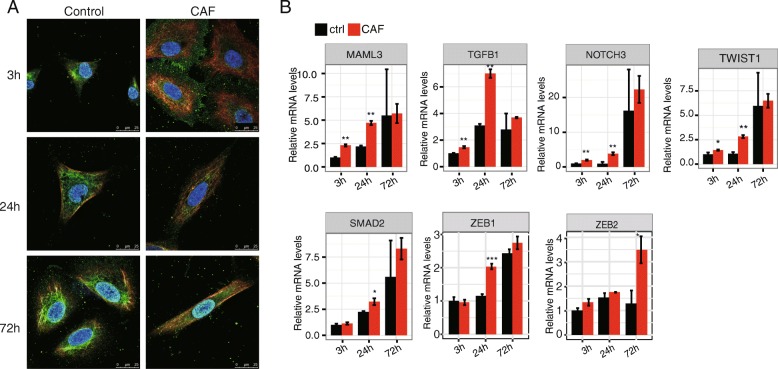
CAF-CM induces expression of mesenchymal markers in A549 cell**. a** Immunostaining of E-cadherin (green), vimentin (red) and DAPI (blue) of CAF treated and control cells. Beginning at 3 h, CAF treated cells showed increased vimentin and decreased E-cadherin expression compared to controls. **b** RT-qPCR of mRNA levels of EMT marker genes in A549 cells with CAF conditioned medium or control treatment at 3, 24 and 72 h. Error bars are SD. *, *p* < 0.05; **, *p* < 0.01; *t* test

We then investigated the expression of mesenchymal markers and found that vimentin was clearly induced in CAF-CM treated cells at3 hours in A549 cells. The induced vimentin expression persisted at 24 and 72 h although at a lower extent (Fig. 2a). As expected, the expression of epithelial marker, E-cadherin, was consistently lower in CAF treated A549 cells, from 3 to 72 h (Fig. 2a). When compared the expression of these two markers, we found the difference between vimentin and E-cadherin was dramatic, which was greatly declined at 72 h of induction.

The expression of other mesenchymal markers (MAML3, NOTCH3, SMAD2, TGFB1, TWIST1and ZEB1/ZEB2) showed a similar trend (Fig. 2b). Except for SMAD2, ZEB1 and ZEB2, we observed the up-regulation of mesenchymal genes as early as 3-h treatment. Except ZEB2, the other six EMT markers showed a significant up-regulation at 24 h, but not at 72 h (Fig. 2b), which indicated a desynchronization between EMT phenotypes and marker gene expression. ZEB2 exhibited upregulation at 72 h (Fig. 2b). The fact that the systematically induced expression of EMT-related genes begins before phenotypical changes compelled us to investigate further into the molecular features of cancer cells at early EMT stages. These results also supported that the in vitro CAF-CM system is effective in inducing A549 EMT and suitable for the analysis of early EMT markers.

### The temporal patterns of differentially expressed genes in CAF-induced A549 EMT

**Fig. 3:**
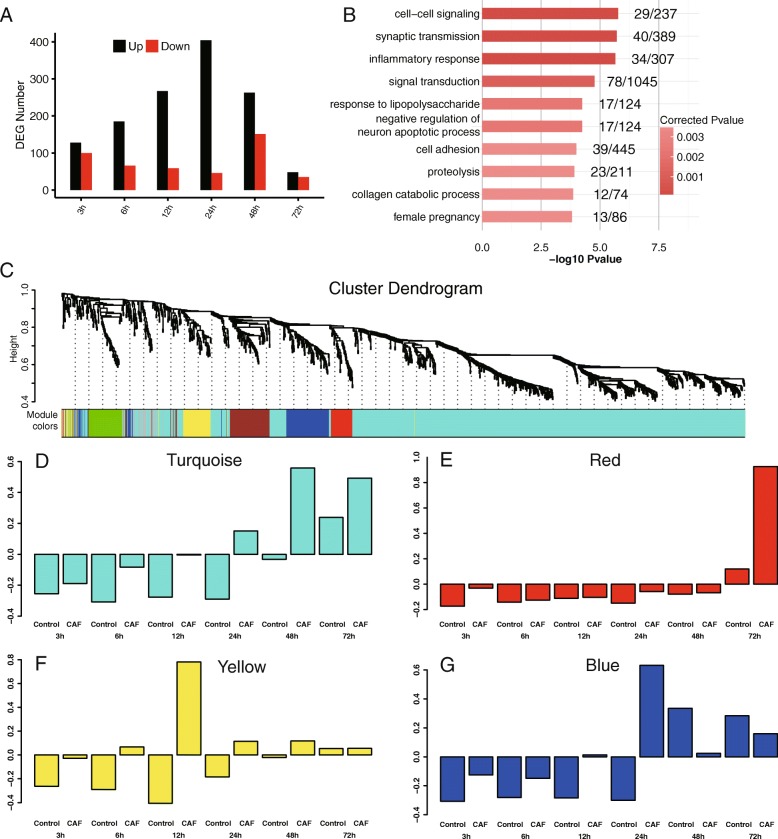
WGCNA analysis of the temporal mRNA expression changes of CAF-CM induced A549 EMT**. a** Number of DEGs at each time point between CAF-CM and control treated cells. **b** GO analysis of the biological process ontology of all DEGs between CAF-CM and control treated cells. Top ten terms and corrected *p*-values were shown. **c** Hierarchical cluster dendrogram of all DEG modules. Modules corresponding to branches are labeled with colors indicated by the color bands underneath the tree. **d**-**g** Eigengene bar plot of turquoise (**d**), red (**e**), yellow (**f**) and blue (**g**) modules

To assess how CAF drives the progression of EMT, we performed RNA-seq on A549 cells at six different time points ranging from 3 to 72 h. We observed a total of 1346 DEGs (*p*-value ≤0.01 and absolute fold change≥2) between CAF-CM treated and control A549 cells at all-time points combined. At 3 h, about a hundred genes were either up- or down-regulated in CAF treated cells, respectively. From 6 to 24 h, up-regulated genes steadily increased to 404, whereas down-regulated genes gradually went down to 46 (Fig. 3a). A sharp shift in the number of deregulated genes occurred at 48 h, when the up-regulated genes declined to 263 and the down-regulated genes increased to 151 (Fig. 3a). A smaller number of genes are de-regulated at 72 h, with 48 and 35 genes getting up- or down-regulated, respectively (Fig. 3a). The sharp difference between 24 and 48 h indicates a physiological change between the early and late stages of induction.

Top terms by Gene Ontology (GO) enrichment analysis of the DEGs contain EMT-related pathways, such as cell-cell signaling, inflammatory response, signal transduction and cell adhesion (Fig. 3b). A couple of terms associated with neuronal functions (synaptic transmission and negative regulation of neuron apoptotic process) are also high on the list (Fig. 3b), which could be related to the morphological changes that A549 cells undergo during EMT.

The results confirmed the conclusion that CAF treatment induces A549 cell EMT, and further suggested that CAF-promoted EMT is characterized by transcriptional change of genes at early stages prior to the appearance of EMT phenotypes, which is consistent with the expression patterns of EMT markers shown in Fig. 2. All six EMT markers were up-regulated upon CAF-induction, although only one of them was DEG (Additional file 1: Figure S1A-F). Interestingly, the epithelial marker gene *CHD1* encoding E-cadherin was only reduced at 3 h of CAF induction but increased afterward (Additional file 1: Figure S1G), indicating the presence of post-transcriptional controls for production of E-cadherin protein.

To further analyze the temporal pattern of gene regulation during CAF induced EMT, we analyzed co-regulated genes between adjacent time points from 3 to 24 h. The overlap of down-regulated genes was very low (Additional file 2: Figure S2A), indicating that during the early hours of EMT progression distinct groups of genes were down-regulated at different time points. Up-regulated genes showed a higher level of overlap, especially towards the later time points (Additional file 2: Figure S2B).

### WGCNA analysis revealed four CAF-induced and two CAF-repressed expression modules

WGCNA was applied to identify module eigengenes (MEs) response to CAF-CM treatment. The1346 DEGs were clustered into seven modules (Fig. 3c). The eigengene bar plots showed that the four modules in the same branch (turquoise, red, yellow and blue) exhibit a time-dependent gene upregulation under the CAF-induced condition (Fig. 3d-g). First, the turquoise module contains the largest number of 932 genes, with a trend of gene upregulation in CAF treated cells from 6 to 72 h. The most drastic upregulation occurs between 24 and 48 h (Fig. 3d). This timing is consistent with the morphological changes and expression of EMT marker genes. GO analysis also showed that these genes are associated with classical EMT pathways, including inflammatory response, cell adhesion and cell-cell signaling (Additional file 7: Table S2). The red module contains genes that are deregulated only at 72 h (Fig. 3e). Judging from the cell morphology and EMT marker expression at this time point, these genes may be responsible for the maintenance of the mesenchymal state of the cells, rather than promoting EMT.

The yellow and blue modules, on the other hand, have shown the trend of gene upregulation at earlier time points. Genes in the yellow module are upregulated in CAF treated cells mainly between 3 and 24 h, and peaked at 12 h (Fig. 3f). The blue module showed a similar trend which peaked at 24 h and reverted at 48 h (Fig. 3g). Genes in these two modules are significantly deregulated at earlier time points and within a smaller window of time, thus we hypothesize that they may contain early EMT markers, which is further analyzed and described below.

The green and brown modules showed a CAF-repressed pattern. Brown module showed a time-dependent repression from 3 to 24 h, whereas green module showed the most pronounced repression at 3 h of CAF-treatment (Additional file 3: Figure S3A). The CAF-downregulated genes were not enriched in any EMT related pathways as expected. The green module was enriched in genes involved in positive regulation of apoptotic process and signal transduction, and the brown module was enriched in genes in DNA-dependent transcription (Additional file 3: Figure S3B).

### Biological pathway analysis of blue and yellow modules reveals early EMT markers

We next explored in more detail the two modules that highlight early gene deregulation during EMT. Functional analysis of the 109 blue module genes resulted in the enrichment of membrane located and transmembrane transport related genes (Additional file 8: Table S3). We noticed that only 10 of them were annotated with a GO biological process term, and six of them enriched in transmembrane transport (Additional file 8: Table S3). A total of 26 genes were enriched in integral to membrane (Additional file 9: Table S4).

**Fig. 4:**
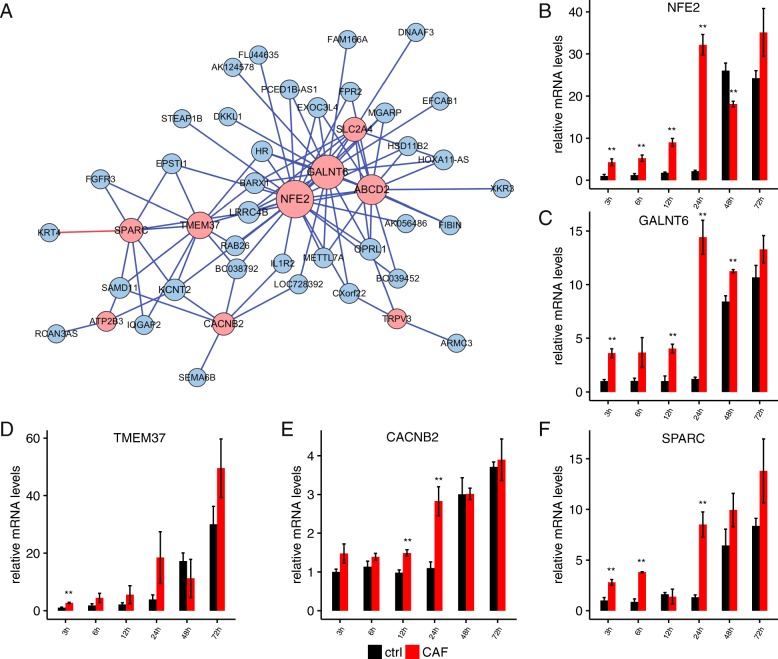
Blue module mRNAs represent a class of early EMT markers. **a** Network connection of the most highly connected genes in the blue module. Blue lines indicate positive expression correlation, and red line indicates negative expression correlation. Pink circles indicate transmembrane transporters in this module. **b**-**f** RT-qPCR results of mRNA levels of key blue module genes in CAF treated and control A549 cells at different time points. Error bars are SD. *, *p* < 0.05; **, *p* < 0.01; *t*-test

A correlation matrix among all 109 genes was generated, at a stringency of *p*-value ≤0.01 and absolute Pearson correlation coefficient abs (PCC) ≥ 0.9, a total of 558 co-expression pairs were detected (Additional file 10: Table S5).Several transmembrane transport genes (GALNT6, ABCD2, SLC2A, TMEM37 and TRPV3) were also regulatory hubs within the blue module (Fig. 4a, shown in pink). In addition, three genes NFE2, SPARC, ATP2B3 in blood coagulation were also regulatory hubs. Consistent with RNA-seq results, the five genes validated by RT-qPCR (Fig. 4b-f) have shown a clear trend of upregulation in CAF treated cells from 3 to 24 h; while TMEM37 is observed as the most significant upregulated gene at 24 h.

**Fig. 5:**
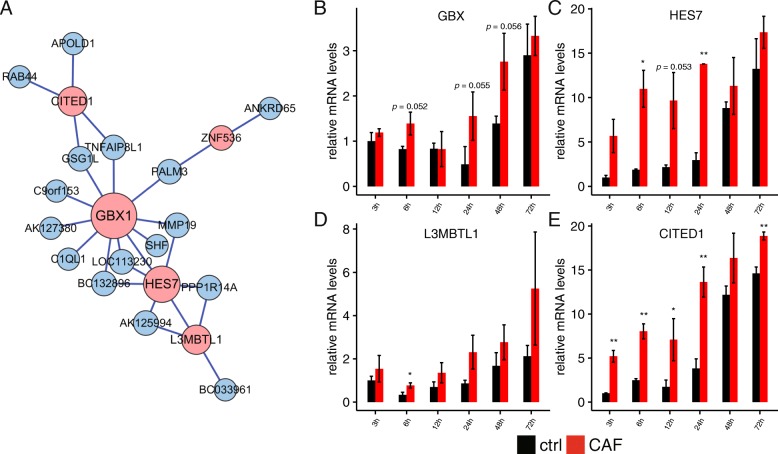
Characteristics of the yellow module mRNAs during early EMT. **a** Network connection of the most highly connected genes in the yellow module. Blue lines indicate a positive expression correlation, and red line indicates a negative expression correlation. Pink circles indicate transcription regulatory genes within the module. **b**-**f** RT-qPCR results of mRNA levels of key yellow module genes in CAF treated and control A549 cells at different time points. Error bars are SD. *, *p* < 0.05; **, *p* < 0.01; *t*-test

Similar analysis was performed on the yellow module. Six genes GBX1, ZNF536, L3MBTL1, ZNF391, CITED1 and HES7 had GO annotation and they were all involved in DNA-dependent transcription and transcription regulation (Additional file 8: Table S3). Network analysis has also put the transcription factors GBX, HES7, L3MBT-1 and CITED1 in the hub positions (Fig. 5a). The expression patterns of these four hub genes tested by RT-qPCR (Fig. 5b-e) have shown the CAF-induced upregulation from 3 to 24 h.

These data highlighted several classes of early EMT markers, the transmembrane transporters and blood coagulation in the blue module, and the transcription factors in the yellow module. Considering that expression of the latter was peaked 12 h early than the former, it is possible that these three classes of markers are separately regulated and underline separable and interconnected EMT promoting mechanisms. For example, the early EMT transcription factors could drive the induction of the transmembrane transporters and blood coagulation.

### miR-3613 regulates EGFR pathway during early EMT

**Fig. 6:**
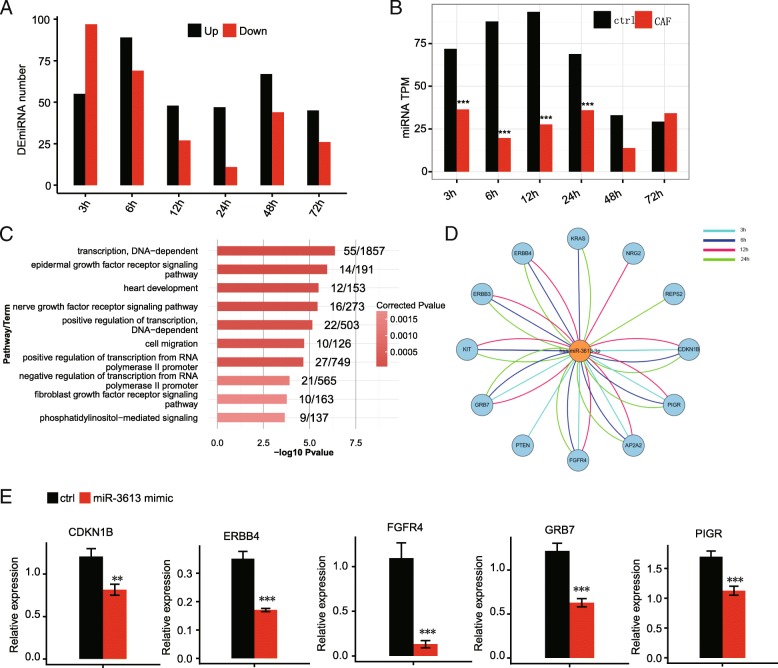
MiR-3613 regulates EGFR pathway genes in CAF induced A549 early EMT**. a** Number of DEmiRNAs at each time point between CAF and control treated A549 cells. **b** Transcripts per million (TPM) of miR-3613-3p in CAF treated and control A549 cells. ***, *p* < 0.001, Fisher’s exact test. **c** GO analysis of the biological process ontology of miR-3613 predicted targets. Top ten terms and corrected *p*-values were shown. **d** Predicted miR-3613-3p regulatory targets in the EGFR pathway from 3 to 24 h after CAF treatment. **e** Barplot showing the downregulation of miR-3613 targets after miR-3613 overexpression. **, *p* < 0.01, ***, *p* < 0.001; t-test

To gain a more comprehensive view of EMT transcriptome, we performed miRNA-seq on the CAF-CM treated A549 cells and controls. We identified differentially expressed miRNAs (DEmiRNA) at each time point (*p* < 0.01 and absolute fold change > 2) (Fig. 6a, Additional file 11: Table S6). More DEmiRNAs were detected at the early stages (3 h and 6 h) of this progress (Fig. 6a). We asked whether the DEmiRNAs showed any temporal trend of de-regulation similar to DEGs from RNA-seq. The numbers of co-regulated miRNAs between adjacent time-points were quite low (Additional file 4: Figure S4A-B). No miRNA was up-regulated at all time-points from 3 to 24 h, and only has-miR-3613-3p was found to be consistently down-regulated (Fig. 6b).

We then performed functional analysis on the predicted target genes of miR-3613 (Fig. 6c). In the biological process, the second highest term was the EGFR pathway, which has been shown to be activated to promote EMT [40]. Given the target gene expression changes, we plotted out the potential regulatory network between miR-3613 and its EGFR pathway targets at different time points (Fig. 6d). Starting as early as 3 h of CAF treatment, miR-3613 is steadily down-regulated during the early progression of A549 cell EMT, which potentially regulates a number of known EMT regulator genes throughout the process.

To further explore the impacts of miR-3613 on EGFR pathway, we transfected the miR-3613 mimic into A549 cells to elevate the cellular level of miR-3613 (Additional file 4: Figure S4C). Seven miR-3613 target genes in EGFR signaling pathway were selected to check their expression after miR-3613 mimic transfection. Five out of the seven genes, including CDKNB1, ERBB4, FGFR4, GRB7, and PIGR, showed significantly down regulation after miR-3613 overexpression (Fig. 6e), implying that miR-3613 play important roles in EGFR signaling regulation. We propose that miR-3613 may serve as an early miRNA marker for EMT, although further studies are required to further pursue miR-3613 regulation.

### The expression pattern of early EMT hallmark genesGALNT6, SPARC and HES7 in vitro is recaptured in LUAD TCGA samples

**Fig. 7:**
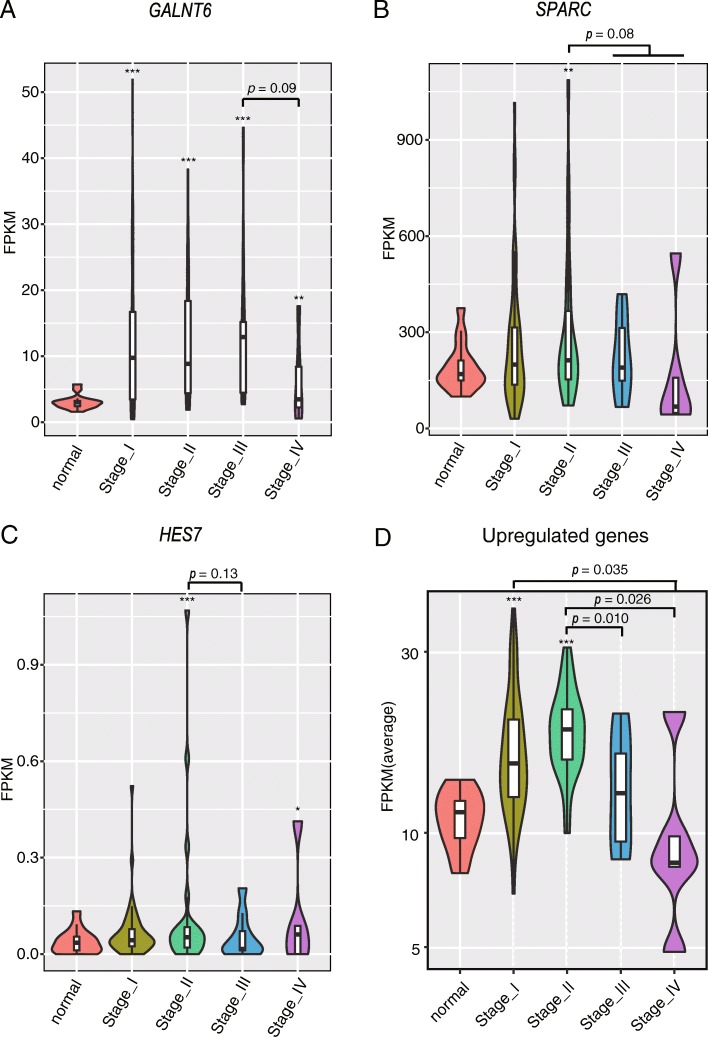
GALNT6, SPARC and HES7 are up-regulated in early stage LUAD**.** Violin plot showing GALNT6 (**a**), SPARC (**b**) and HES7 (**c**) mRNA levels in normal lung tissue and lung adenocarcinoma using RNA-seq data from TCGA database. (**d**) Violin plot showing the average expression level of genes with elevated expression pattern at early stages (I and II) LUAD from brown module. *, *p* < 0.05; **, *p* < 0.01; ***, *p* < 0.001; compared to normal tissues or as indicated

To evaluate the clinical relevance of potential early EMT hallmark genes, we analyzed their mRNA expressions in LUAD dataset from TCGA. Since results from A549 cell lines showed that these genes are up-regulated mainly during early EMT events, we broke down the mRNA expressions by clinical stages, and hypothesized that these genes would be more severely over-expressed at early cancer stages (stage I and II) than advanced stages (stage III and IV).We examined the expression patterns of nine early EMT hallmark genes in the clinical samples. Strikingly, three were consistent with the predicted pattern for EMT early markers. GALNT6 was significantly up-regulated in tumor tissues in all stages, but the trend of up-regulation showed a decline from stage III to stage IV LUAD patients (Fig. 7a). The mRNA expressions of SPARC and HES7 only increased in stage II tumors compared to normal tissues, and their stage II expressions are higher than that of later stage tumors (Fig. 7b and c). We then analyzed the global expression pattern of genes up-regulated from 3 to 24 h by WGCNA method, and found that the brown module containing 69 genes showed elevated expression pattern at early stages (I and II) of LUAD (Fig. 7d). The dynamic expression of the early EMT hallmark genes GALNT6, SPARC and HES7 during LUAD progression, higher expression in early stages I and II and lower in advanced stages III and IV, suggest their biological roles could be stage-specific.

## Discussion

In this study, we described an in vitro cell-culture model that mimics EMT of A549 cells by induction of CAF-CM that was produced by human fetal lung fibroblast WI-38 cells activated by A549 cell culture medium. This model is easy to operate and can be robustly repeated, and has been repeated in another lung cancer cell (PC9) [41]. High-throughput sequencing revealed a time-dependent upregulation of genes from two WGCNA modules; one peaked at 12 h and the other at 24 h of induction. In both cases, the induced expression dropped after the peak stages. These two modules of genes are highly enriched in transmembrane transport, blood coagulation, and transcription regulation. We then analyzed the expression of nine of the annotated genes in LUAD patient samples obtained by TCGA, and found three of them were specifically upregulated in cancer at early but not advanced LUAD stages.

Molecular profiling of clinical specimens has provided abundant information on diagnostic biomarkers and therapeutic targets of cancer in EMT research [42–45]. However, due to the limitations of sample collection and time points, current EMT markers mainly focus on late stages when the EMT has already been accomplished. Besides, clinical findings were unable to be applied for further study in the same system. However, cell models were the optimal choices as they are easy to operate. Different from previous successful cases of EMT models [25, 27, 28], our cell-based EMT model utilizing CM of cultured CAFs from in vitro cultured cells was a reliable system allowing assessment of similarities and differences between the cell line and primary human lung cancer.

In the blue module, two transmembrane proteins together playing a central role in the regulatory network are involved in cancer metastasis. GALNT6 has been implicated in the metastasis of multiple cancers [46, 47]. It is upregulated in pancreatic cancer cells, and its silence reduces the level of EGFR2 and cell viability [46]. Besides, SPARC is a secreted matricellular protein governing cell adhesion, proliferation and differentiation, and driving pathological responses in non-small cell lung cancer [48]. SPARC may also serves as an unfavorable prognostic marker in pancreatic cancer, as its overexpression may improve cell invasion [49].

The yellow module revealed transcription regulators involved in EMT initiation. CITED1 is the most well-studied in melanoma progression that can be activated by the TGFβ-SMAD2 pathway and promote amoeboid migration of melanoma cells [50]. Intriguingly, this metastatic behavior is distinct from EMT, thus making us wonder whether the same or a different pathway is involved in CITED1 regulated EMT. Another bHLH transcription factor HES7regulated by Notch signaling pathway [51] has been shown to express in cervical cancer [52], and its roles in cancer progression is unclear.

Many key EMT transcription factors are under miRNA regulation, such as the metastasis suppressive function of miR-200 family in targeting the TGFβ/ZEB pathway [53, 54]. Our data have revealed another potential EMT regulatory pathway, in which the miR-3613 regulation of EGFR pathway may contribute to the promotion of EMT by CAF conditioned medium. EGFR activation has been proven to promote cancer cell proliferation, EMT and drug resistance [40, 55–57], and multiple EGFR pathway genes have been proposed as anti-metastatic drug targets. MiR-3613 was recently identified to overexpress in ovarian cancers and down-regulate PTEN [58], a regulator of PI3K-Akt signaling downstream of EGFR. The regulation between miR-3613 and its potential targets may present novel therapeutic targets to overcome drug resistance caused by EGFR mutation (Additional file 5: Figure S5).

Generally, this study presents a new reliable in vitro model for CAF-induced EMT, which is supported not only by the cell morphology and EMT markers, but also by identification of classic EMT-related functional pathways. Moreover, this model allows a time-dependent monitoring of the EMT progress, which led to the identification of the early EMT hallmark genes. Strikingly, three early EMT hallmark genes GALNT6, SPARC and HES7 show the similar stage-specific expression pattern in LUAD TCGA samples, which can be further studied for diagnosis of early stages of lung cancer and for developing anticancer drugs (Additional file 5: Figure S5). Furthermore, the cell-model could facilitate the future studies for screening additional biomarkers and cell-based drugs. This study proves that a combination of the cell-based study and the available patient genome-wide data can greatly expedite the translational medicine process.

## Conclusions

This study presented a reliable cell-based EMT model and several classes of novel early EMT markers identified by this model. Three of the early EMT markers were confirmed by TCGA LUAD transcriptome data. Results from the combination of cell-based screening and patient data validation introduce new prognostic markers and therapeutic targets for LUAD, as well as a cell-based model ready for studying their mechanisms of action and for drug screening.

## Additional files

Additional file 1: **Figure S1.** Temporal mRNA expression changes of CAF induced A549 EMT. (A-G) Bar plots show expression levels of EMT marker genes in A549 cells with CAF conditioned medium or control treatment. *, 0.01 < *p* < 0.05; **, *p* < 0.01; ***, *p* < 0.001. (PDF 908 kb)

Additional file 2: **Figure S2.** Temporal gene expression changes of CAF induced A549 EMT. Venn diagrams of DEGs at 3, 6, 12 and 24 h. Numbers of down-regulated (**A**) and up-regulated (**B**) genes at each time point were shown. (PDF 2195 kb)

Additional file 3: **Figure S3.** Expression and functionprofile of eigengenemodules**.** (**A**) Expression pattern of genes represented in brown and green modules. (**B**)GO biological processes enriched with genesbelonging to brown and greenmodules. (PDF 928 kb)

Additional file 4: **Figure S4.** Temporal miRNA expression changes of CAF induced A549 EMT**.** Venn diagrams of DEmiRNAs at 3, 6, 12 and 24 h. Numbers of down-regulated (**A**) and up-regulated (**B**) miRNAs at each time point were shown. (**C**) Bar plot showing the RT-qPCR results of miR-3613 mimic experiments in A549 cells. (PDF 2329 kb)

Additional file 5: **Figure S5.** Proposed model figure of the main methods and findings in this study**.** Early EMT markers including GALNT6, SPARC and HES7 showed elevated expression level at early stages of CAF-CM induction. Downregulation of miR-3613 may also promotes EMT by releasing the EGFR signaling pathway genes. (PDF 189 kb)

Additional file 6: **Table S1.** List of primers used for RT-qPCR. (XLSX 11 kb)

Additional file 7: **Table S2.** Top 10 GO biological process terms and pathways of genes from turquoise module. (XLSX 10 kb)

Additional file 8: **Table S3.** GO biological process terms and pathways of genes from Blue and Yellow module genes. (XLSX 10 kb)

Additional file 9: **Table S4.** GO cellular components enriched with genes belonging to blue module. (XLSX 10 kb)

Additional file 10: **Table S5.** Correlation pairs statistics and the corresponding pairs in each module. (XLSX 9 kb)

Additional file 11: **Table S6.** The list of DEmiRNAs between CAF and control from 3, 6, 12, 24, 48, 72 h samples. (XLSX 2789 kb)

## Data Availability

The data discussed in this publication are available under GEO Series accession number GSE90133.

## Abbreviations

CAF: Cancer-associated fibroblast
CM: Conditioned medium
DEG: Differentially expressed genes
DEmiRNA: Differentially expressed miRNA
EMT: Epithelial-mesenchymal transition
GO: Gene Ontology
WGCNA: Weighted gene coexpression network analysis
