# Supplementary figures and images for: Epithelial-mesenchymal transition markers screened in a cell-based model and validated in lung adenocarcinoma
Source: BMC Cancer. 2019 Jul 11;19:680. doi: 10.1186/s12885-019-5885-9 (PMC6624955; doi:10.1186/s12885-019-5885-9)

Fig. S1

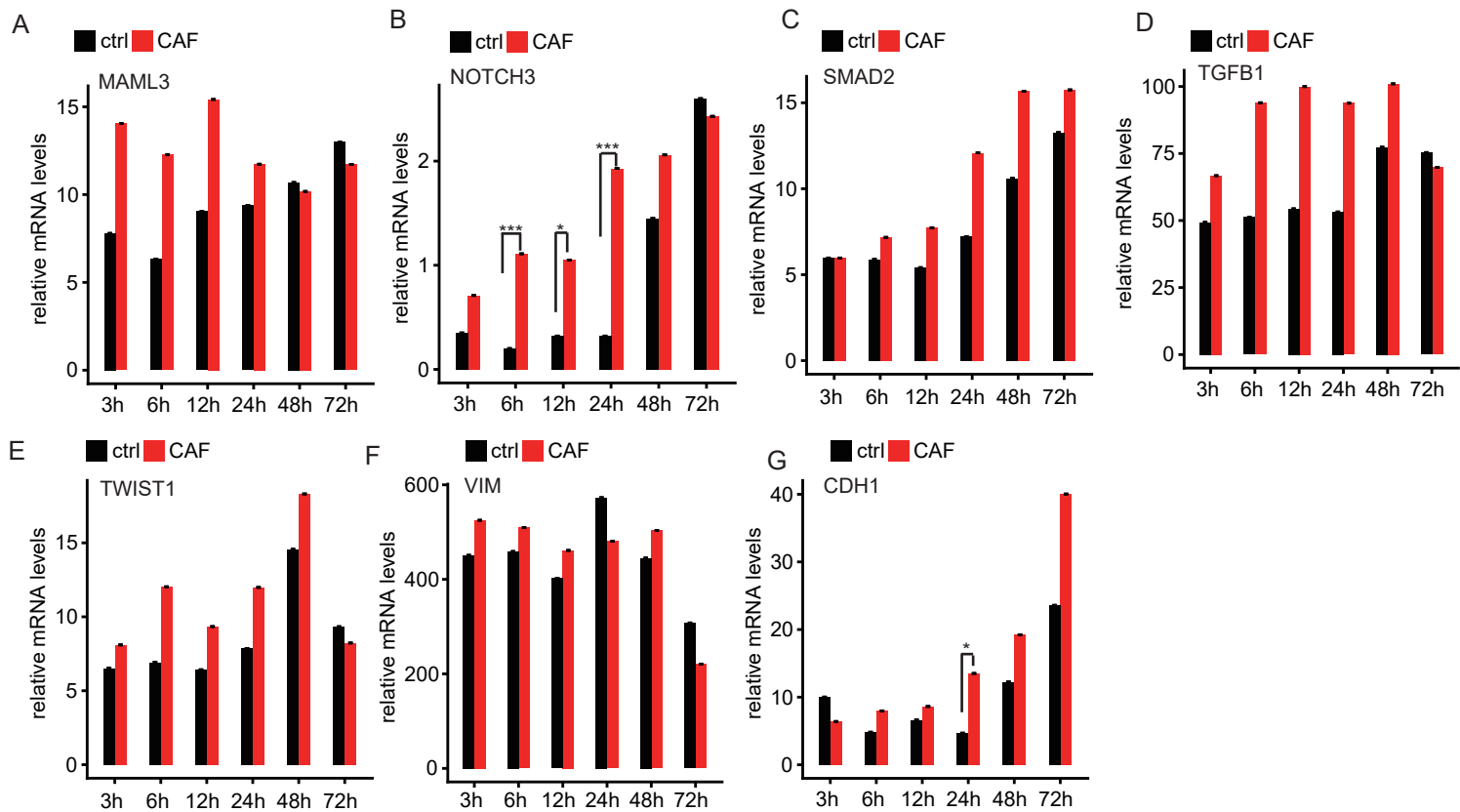

Supplement: Supplementary file 1 — Figure S1. Temporal mRNA expression changes of CAF induced A549 EMT. (A-G) Bar plots show expression levels of EMT marker genes in A549 cells with CAF conditioned medium or control treatment. *, 0.01 < p < 0.05; **, p < 0.01; ***, p < 0.001. (PDF 908 kb) [file 12885_2019_5885_MOESM1_ESM.pdf]

A

Downregulated Genes

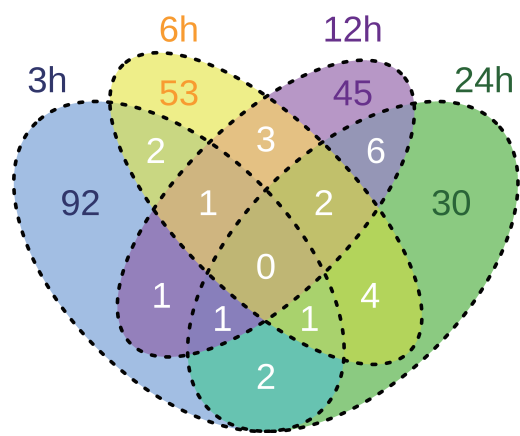

B

Upregulated Genes

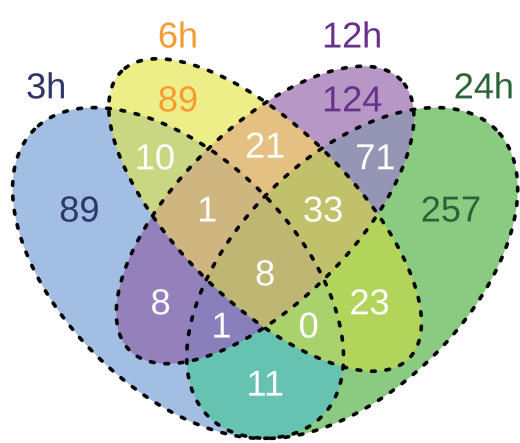

Supplement: Supplementary file 2 — Figure S2. Temporal gene expression changes of CAF induced A549 EMT. Venn diagrams of DEGs at 3, 6, 12 and 24 h. Numbers of down-regulated (A) and up-regulated (B) genes at each time point were shown. (PDF 2195 kb) [file 12885_2019_5885_MOESM2_ESM.pdf]

Fig. S3

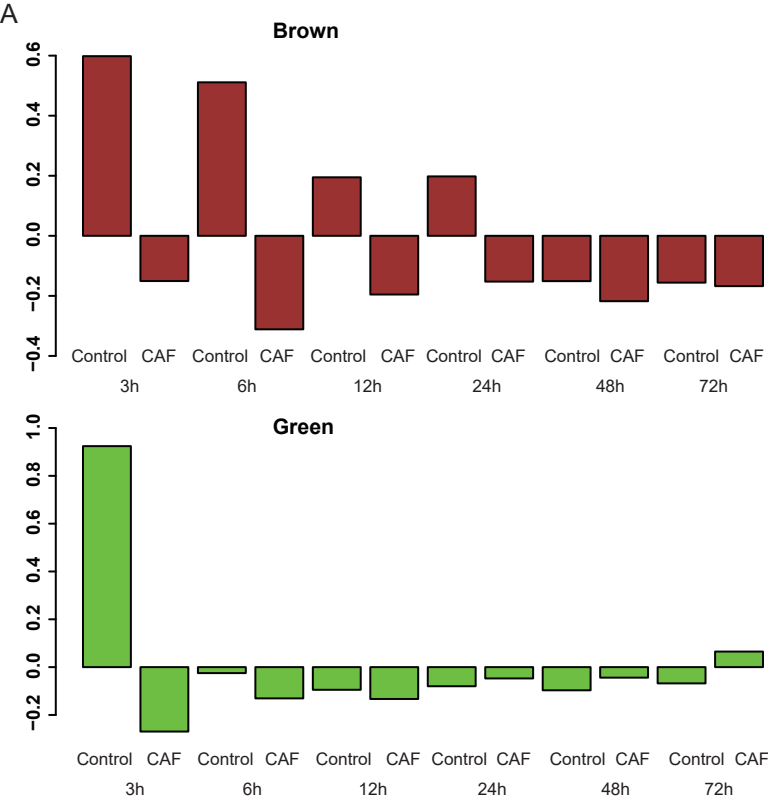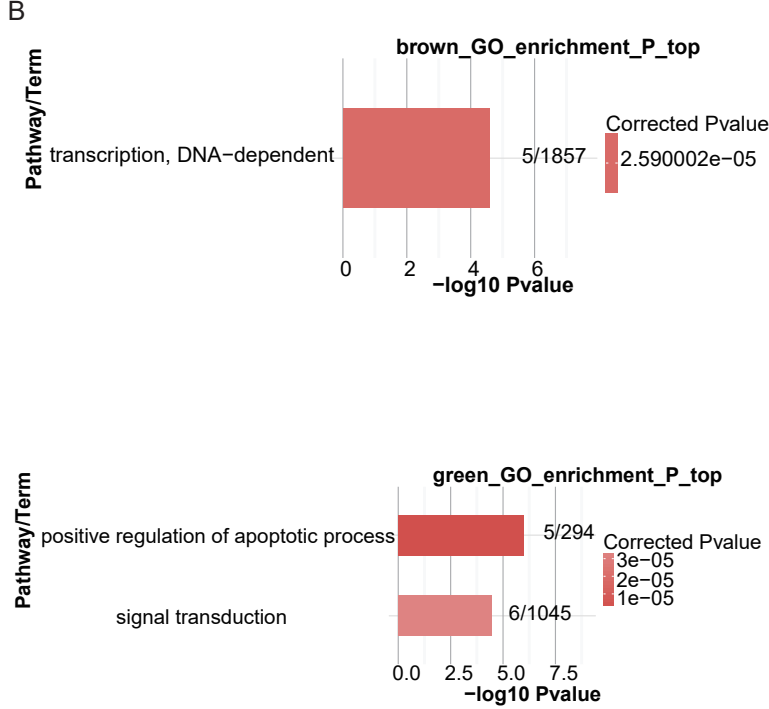

Supplement: Supplementary file 3 — Figure S3. Expression and functionprofile of eigengenemodules. (A) Expression pattern of genes represented in brown and green modules. (B)GO biological processes enriched with genesbelonging to brown and greenmodules. (PDF 928 kb) [file 12885_2019_5885_MOESM3_ESM.pdf]

Fig. S4

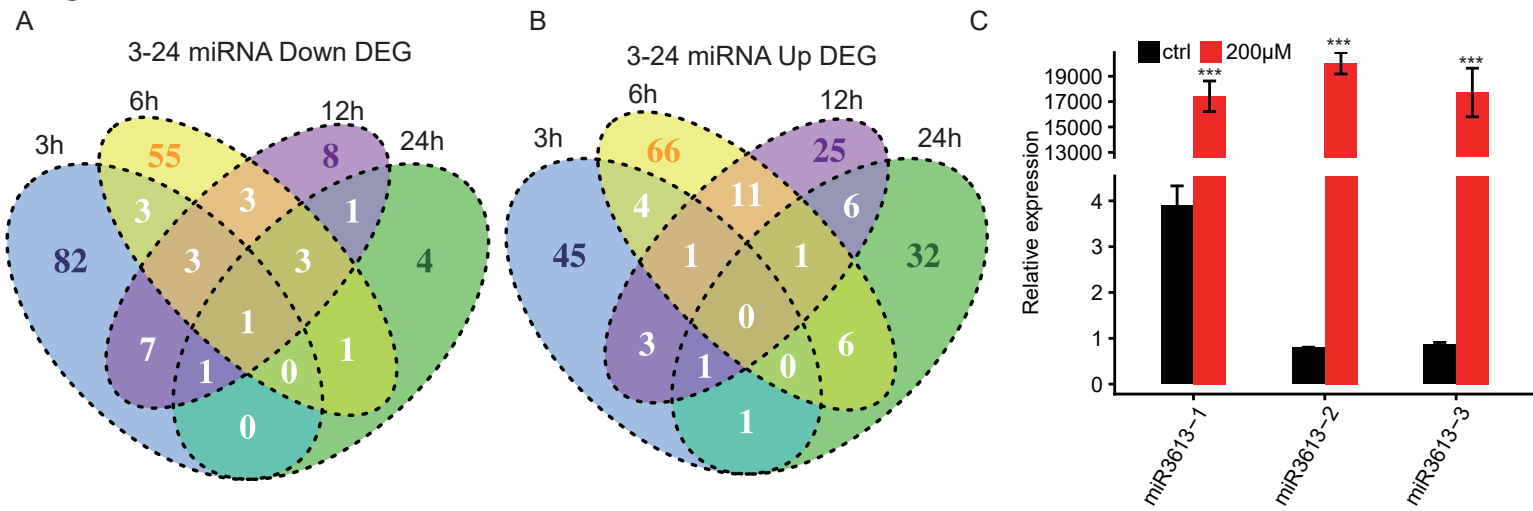

Supplement: Supplementary file 4 — Figure S4. Temporal miRNA expression changes of CAF induced A549 EMT. Venn diagrams of DEmiRNAs at 3, 6, 12 and 24 h. Numbers of down-regulated (A) and up-regulated (B) miRNAs at each time point were shown. (C) Bar plot showing the RT-qPCR results of miR-3613 mimic experiments in A549 cells. (PDF 2329 kb) [file 12885_2019_5885_MOESM4_ESM.pdf]

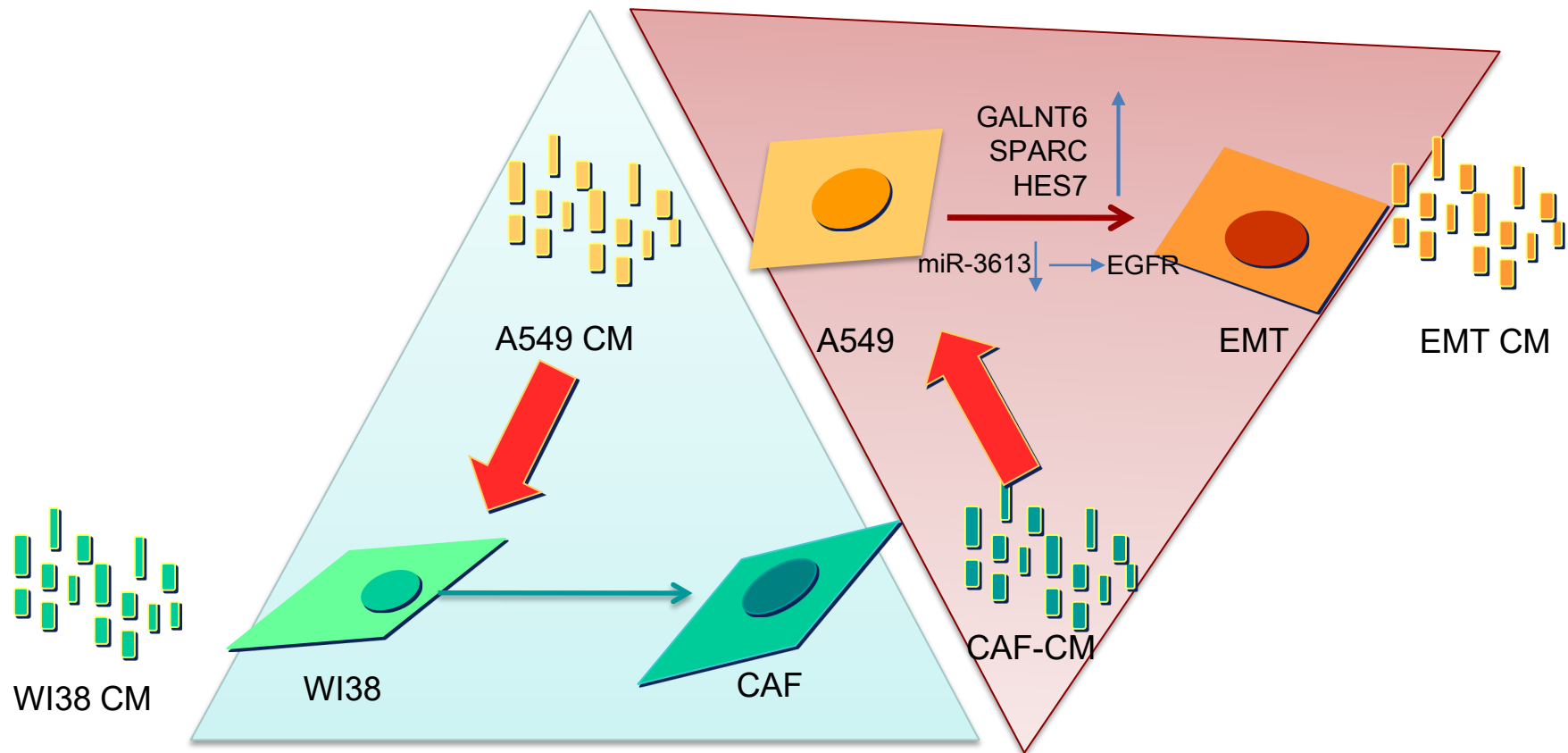

Supplement: Supplementary file 5 — Figure S5. Proposed model figure of the main methods and findings in this study. Early EMT markers including GALNT6, SPARC and HES7 showed elevated expression level at early stages of CAF-CM induction. Downregulation of miR-3613 may also promotes EMT by releasing the EGFR signaling pathway genes. (PDF 189 kb) [file 12885_2019_5885_MOESM5_ESM.pdf]
